# Computational design and development of high-performance polymer-composites as new encapsulant material for concentrated PV modules

**DOI:** 10.1038/s41598-020-62191-9

**Published:** 2020-03-24

**Authors:** Kabeer Raza, Syed Sohail Akhtar, Abul Fazal M. Arif, Abbas Saeed Hakeem

**Affiliations:** 1 Mechanical Engineering Department, King Fahd University of Petroleum & Minerals (KFUPM), Dhahran, Saudi Arabia; 2 Center of Excellence in Nanotechnology, King Fahd University of Petroleum & Minerals, Dhahran, Saudi Arabia; 3 Department of Mechanical Engineering, McMaster Manufacturing Research Institute, McMaster University, Hamilton, Canada

**Keywords:** Composites, Mechanical properties, Computational methods

## Abstract

A computational design methodology is reported to propose a high-performance composite for backside encapsulation of concentrated photovoltaic (CPV) systems for enhanced module life and electrical power. Initially, potential polymer composite systems that are expected to provide the target properties, such as thermal conductivity, coefficient of thermal expansion, and long-term shear modulus are proposed using in-house built design codes. These codes are based on differential effective medium theory and mean-field homogenization, which lead to the selection of matrix, filler, volume fractions, and type of particulates. Thermoplastic polyurethane (TPU) loaded with ceramics fillers of a minimum spherical diameter of 6 μm are found potential composites. Some representative samples are synthesized through the melt-mixing and compression-molding route and characterized. The target properties including thermal conductivity, coefficient of thermal expansion, viscoelastic parameters, and long-term shear modulus are measured and used to evaluate the performance of CPV modules using previously published finite element model. The proposed composite can drag the cell temperature down by 5.8 °C when compared with neat TPU which leads to a 4.3% increase in electrical power along with a reasonable module life. It is expected that this approach will make a baseline for the effective production of polymer composites in various industrial applications.

## Introduction

The motive behind the development of any composite material is always to enhance the overall performance of a system, which is directly linked to the intrinsic properties of the composite constituents. For example, a combination of high thermal conductivity (TC) and low coefficient of thermal expansion (CTE) is required for the substrate material in the thermal management of microelectronics^1,2^. For some other applications, the required properties depend on the functions and conditions of the material in focus. The composite attributes such as type of matrix and fillers, their volume fractions, particle size, etc. are selected based on a particular set of target properties for enhanced performance of intended application^3,4^.

The bulk properties of composite materials are complex functions of their constituents at atomic and microstructural levels. The selection of exact constituents which can lead to the target properties can be very tedious and time consuming without using computational tools. Significant efforts are going on to develop computational tools that can predict the constituents at atomic and microstructural levels for multifunctional materials with tailorable bulk properties. Numerous contributions have been made by the “Materials Genome Initiative (GMI)” to accelerate the discovery of new materials by tightly-integrated efforts in experiment, theory and computation^5^. The theories and computational models for the maximization of material performance are evolving very fast owing to the increased available computational power. For example, thermal and electrical transport in thermoelectric material can be optimized via computational models to enhance thermoelectric performance^6^. Similarly, molecular dynamics and continuum mechanics-based models have been used to explore atomic-scale deformation mechanisms in self-folding graphene reinforced composites, and to optimize the self-folded length and the interface for enhancing ductility without losing strength^7^. Similarly, the strengthening mechanisms in polymer blends and nanocomposites have been explored through molecular dynamics simulations^8^ and compared to the experimental results and predictions of micromechanics models^9^. Remarkable improvement in the mechanical properties has been reported with a 5.66 vol% addition of silica nanoparticles owing to an increased density of intermolecular hydrogen bonds^8,9^. The development of material design models to improve magnetoelectric coupling in magnetoelectric composites is also a fast-evolving field for energy harvesting^10–12^. Whereas, another way of computational material design is to used data-driven machine learning^13,14^.

The type of computational design and simulation method depends upon the length and time –scales at which the properties are required to be controlled^15^. Molecular dynamics simulations and continuum mechanics-based models are used for the cases where properties are controlled at atomic/molecular levels^7,16^. Whereas, finite element models^17–20^ and homogenization schemes^17,21–23^ are used to predict the properties which are controlled at the microstructural level. The purpose of the current work is to present an approach for the design and development of composite materials for the backside encapsulant of CPV systems.

Hasan *et al*.^24^ have compared the structural performance of different encapsulants currently used in the PV industry. The performance criteria were light transmittance, UV durability, electrical insulation, moisture ingression, structural life, and cost. They demonstrated that among five different encapsulant materials, ionomer is the best and ethylene-vinyl acetate (EVA) stands at the second position followed by Polydimethylsiloxane (PDMS), thermoplastic polyurethane (TPU) and Polyvinyl butyral (PVB), respectively. However, EVA is more popular in the solar industry due to its relatively lower cost as compared with ionomer.

The main objective of the encapsulant is to electrically insulate the circuit of solar cells. Besides, it also protects the solar cells from environmental effects, electrical leakage, and mechanical damage. To protect from environmental effects, the encapsulant should be resistant to water penetration and yellowing caused by UV-rays. To avoid leakage of current, the encapsulant must be a good electrical insulator i.e. high volume resistivity is required. Moreover, low strength and low elastic modulus are desired to minimize thermal stresses on the surrounding components. A low CTE is also desired to reduce the hysteresis of interfacial stresses in thermal cycling. Furthermore, the encapsulant has to be transparent from the front side so that maximum solar radiation can be transmitted to the cells with minimum absorbance and reflection^25^.

As compared with non-concentrated systems, the CPV systems encounter higher thermal stresses due to high and irregular distribution of flux and temperature^26^ which leads to overheating and premature breaking of cells and interconnects mainly due to thermal fatigue^24^. Therefore, the requirement of high TC, low elastic modulus and low CTE becomes vital for the continued efficient performance of CPV systems. High TC can help the cells cool faster while low elastic modulus and coefficient of thermal expansion can reduce the thermal stresses in the encapsulated components. The authors have previously conducted a performance evaluation of the CPV system using a finite element model to set the target TC, long-term shear modulus (*G*_∞_) and CTE considering EVA as a datum line. The heat transfer and thermal stress/strain problems were sequentially coupled and solved with variable input material properties of backside encapsulant to evaluate the performance (electrical power and module life) in each parametric study. Finally, it was reported that a TC ≥ 0.75 Wm^−1^K^−1^, CTE ≤ 200 × 10^−6^ K^−1^, and *G*_∞_ ≤ 0.2 MPa of backside encapsulant in the CPV module is required for enhanced electrical power and module life^27^.

In this work, composite material as an encapsulant for the backside of CPV systems is designed at the microstructural level with target levels of thermal and structural properties based on the authors’ previous work^27^. The complete design scheme and its implementation are explained. The phases, compositions, particle size and other material attributes are predicted using design codes to acquire the target properties. Some representative composite samples are synthesized in line with the predictions and the resulting properties are measured for validation. The viscoelastic properties of the samples are also estimated for performance evaluation. The performance evaluation of the developed composite systems is conducted through a finite element model by taking into consideration a representative three-cell PV laminate with measured properties and constitutive behavior as inputs.

## The scheme for the Computational Design of Composite Materials

Finding the critical material properties and setting the target range is the first step of the computational design scheme. The material properties, which are sensitive for improved performance, can be called as ‘critical material properties’. The problem of how the critical material properties are affecting the performance can be solved by analytical or numerical methods. The analytical or numerical solution of a problem solved with different combinations of material properties would elucidate their effects and sensitivity for the performance of the system and hence would lead to set the target range of the material properties. The second step is to design the material i.e. to select the material attributes such as the phases, their volume fractions, particle size, distribution and surface condition, etc. One of the solution strategies is to use property estimation models to predict the best combinations of material attributes for the intended properties. To implement this strategy, initially, the possible phases including both matrices and fillers are identified such that their combinations should not violate any major requirement. The essential requirement for any encapsulant material is to provide electrical insulation to the cells and hence the selected potential phases in the indented composite should have high volume resistivity. To meet this requirement, the best matrix is selected from the available candidates so that the loading of potential fillers leads to achieving the target material properties. In the next step of material design, the potential fillers, their required volume fractions, appropriate particle size, shape morphology, and distribution are selected by solving equations of material properties estimation models. Some theoretical models used in this work are described in the following section.

### Elastic modulus and coefficient of thermal expansion (CTE)

Akhtar *et al*.^3^ have found a good agreement between the experimentally measured CTE and the one predicted by mean-field homogenization of Mori-Tanaka as outlined by Benveniste and Dvorak^28^. According to the mean-field homogenization scheme the effective properties are estimated using the Eqs. (1) to (3).

1 $$\overline{{C}_{eff}}={\varphi }_{f}{C}_{f}\,:{A}_{f}+(1-{\varphi }_{f}){C}_{m}\,:{A}_{m}$$

2 $${\alpha }_{eff}={\alpha }_{f}{I}_{2}+{\varphi }_{f}({C}_{f}^{-1}-{C}_{m}^{-1})W{[(1-{\varphi }_{f}){I}_{4}+{\varphi }_{f}W]}^{-1}{({C}_{f}^{-1}-{C}_{m}^{-1})}^{-1}({\alpha }_{f}{I}_{2}-{\alpha }_{m}{I}_{2})$$

3 $$\begin{array}{c}{A}_{m}={[(1-{\varphi }_{f}){I}_{4}+{\varphi }_{f}{B}_{a}]}^{-1}\\ {A}_{f}={B}_{a}\,:{A}_{m}\\ {B}_{a}={[{I}_{4}+S:{C}_{m}^{-1}({C}_{f}-{C}_{m})]}^{-1}\end{array}$$

$\overline{{C}_{eff}}$ and ${\alpha }_{eff}$ are the homogenized stiffness tensor and the effective CTE of the resulting composite, respectively whereas $C$ and $\alpha$ represent stiffness tensors and CTE, respectively. $W={C}_{f}{A}_{f}{C}_{m}^{-1}$, ${A}_{m}$ and ${A}_{f}$ are the strain localization tensors, *I*_2_ and *I*_4_ are the 2^nd^ order and 4^th^ order identities respectively, and $S$ is the Eshelby’s tensor.

### Thermal conductivity (TC)

Siddiqui *et al*.^29^ have presented their generalized effective medium theory for dilute hybrid fillers as stated in Eqs. (4) to (13).

4 $${k}_{eff,11}={k}_{eff,22}={k}_{m}\frac{2+\mathop{\sum }\limits_{i=2}^{N}{\varphi }^{i}[{\beta }_{11}^{i}(1-{L}_{11}^{i})(1+{\langle {\cos }^{2}\theta \rangle }^{i})+{\beta }_{33}^{i}(1-{L}_{33}^{i})(1-{\langle {\cos }^{2}\theta \rangle }^{i})]}{2-\mathop{\sum }\limits_{i=2}^{N}{\varphi }^{i}[{\beta }_{11}^{i}{L}_{11}^{i}(1+{\langle {\cos }^{2}\theta \rangle }^{i})+{\beta }_{33}^{i}{L}_{33}^{i}(1-{\langle {\cos }^{2}\theta \rangle }^{i})]}$$

5 $${k}_{eff,33}={k}_{m}\frac{1+\mathop{\sum }\limits_{i=2}^{N}{\varphi }^{i}[{\beta }_{11}^{i}(1-{L}_{11}^{i})(1-{\langle {\cos }^{2}\theta \rangle }^{i})+{\beta }_{33}^{i}(1-{L}_{33}^{i}){\langle {\cos }^{2}\theta \rangle }^{i}]}{1-\mathop{\sum }\limits_{i=2}^{N}{\varphi }^{i}[{\beta }_{11}^{i}{L}_{11}^{i}(1-{\langle {\cos }^{2}\theta \rangle }^{i})+{\beta }_{33}^{i}{L}_{33}^{i}{\langle {\cos }^{2}\theta \rangle }^{i}]}$$

6 $${\langle {\cos }^{2}\theta \rangle }^{i}=\frac{\int {\rho }^{i}(\theta ){\cos }^{2}\theta \,\sin \,\theta d\theta }{\int {\rho }^{i}(\theta )\sin \,\theta d\theta }$$

7 $${L}_{11}^{i}={L}_{22}^{i}=\{\begin{array}{c}\frac{{({p}^{i})}^{2}}{2({({p}^{i})}^{2}-1)}-\frac{{p}^{i}}{2{({({p}^{i})}^{2}-1)}^{1.5}}{\cosh }^{-1}\,{p}^{i},\,\text{for}\,{p}^{i}\ge 1\\ \frac{{({p}^{i})}^{2}}{2({({p}^{i})}^{2}-1)}+\frac{{p}^{i}}{2{(1-{({p}^{i})}^{2})}^{1.5}}{\cos }^{-1}\,{p}^{i},\,\text{for}\,{p}^{i} < 1\end{array}$$

8 $${p}^{i}={a}_{3}^{i}/{a}_{1}^{i}$$

9 $${L}_{33}^{i}=1-2{L}_{11}^{i}$$

10 $${\beta }_{kk}^{i}=\frac{{k}_{c,kk}^{i}-{k}_{m}}{{k}_{m}+{L}_{kk}^{i}({k}_{c,kk}^{i}-{k}_{m})}$$

11 $$\begin{array}{c}{k}_{c,11}^{i}=\{\begin{array}{c}{k}_{f}^{i}/(1+{\gamma }_{11}^{i}{L}_{33}^{i}{k}_{f}^{i}/{k}_{m}),\,{\rm{for}}\,{\rm{platelet}}\,{\rm{shaped}}\,{\rm{fillers}}\\ {k}_{f}^{i}/(1+{\gamma }_{11}^{i}{L}_{11}^{i}{k}_{f}^{i}/{k}_{m}),\,{\rm{for}}\,{\rm{other}}\,{\rm{shapes}}\end{array}\\ {k}_{c,33}^{i}=\{\begin{array}{c}{k}_{f}^{i}/(1+{\gamma }_{33}^{i}{L}_{11}^{i}{k}_{f}^{i}/{k}_{m}),\,{\rm{for}}\,{\rm{cylinderical}}\,{\rm{shaped}}\,{\rm{fillers}}\\ {k}_{f}^{i}/(1+{\gamma }_{33}^{i}{L}_{33}^{i}{k}_{f}^{i}/{k}_{m}),\,{\rm{for}}\,{\rm{other}}\,{\rm{shapes}}\end{array}\end{array}$$

12 $${\gamma }_{kk}^{i}=\{\begin{array}{c}{\alpha }_{k}(2+1/{p}^{i}),\,\text{for}\,{p}^{i}\ge 1\\ {\alpha }_{k}(1+2{p}^{i}),\,\text{for}\,{p}^{i} < 1\end{array}$$

13 $${\alpha }_{k}^{i}={R}_{TB}^{i}{k}_{m}/{a}_{k}^{i}$$

${{k}}_{{inc}}^{{i}}$ The superscript *i* relates the parameter to *i*th filler. The other parameters are listed as follows.

**Table:** 

${\varphi }^{i}$	Volume fraction of *i*th filler
${\langle {\cos }^{2}\theta \rangle }^{i}$	Orientation factor of *i*th filler
${p}^{i}$	Aspect ratio of ith filler
${{a}}_{{i}}^{{k}}$	Radius of *i*th ellipsoid shaped filler along *k*th axis
${{R}}_{{TB}}^{{i}}$	Interfacial thermal resistance of *i*th filler with the matrix
${k}_{f}^{i}$	Thermal conductivity of *i*th filler
${k}_{m}$	Thermal conductivity of matrix

For non-dilute filler concentrations (higher ranges of volume fraction), the authors have modified the Eqs. (4) and (5) following the Bruggeman’s differential effective medium theory by integrating the differential form of low volume fraction relationship. Bruggeman’s theory is based on the postulate that for high volume fractions, the fillers can be added incrementally by considering the existing composite as a new matrix for the current increment. Hence, for low volume fractions, the Eq. (4) can be simplified for small *φ* leading Eq. (14) for which the derivation has been included in the Supplementary Information.

14 $${k}_{eff,11}={k}_{eff,22}={k}_{m}\left(1+\mathop{\sum }\limits_{i=2}^{N}\frac{{\varphi }^{i}}{2}[{\beta }_{11}^{i}(1+{\langle {\cos }^{2}\theta \rangle }^{i})+{\beta }_{33}^{i}(1-{\langle {\cos }^{2}\theta \rangle }^{i})]\right)$$

For Eq. (14) using the analogy of Every’s model^30^, its’ differential and integral form for a single filler can be written as Eqs. (15) and (16) respectively.

15 $$dk=\left(\frac{d\varphi }{1-\varphi }\right)\times \frac{k}{2}\times ({\beta }_{11}(1+\langle {\cos }^{2}\theta \rangle )+{\beta }_{33}(1-\langle {\cos }^{2}\theta \rangle ))$$

16 $${\int }_{{k}_{m}}^{{k}_{eff}}\frac{2}{k({\beta }_{11}(1+\langle {\cos }^{2}\theta \rangle )+{\beta }_{33}(1-\langle {\cos }^{2}\theta \rangle ))}dk={\int }_{0}^{\varphi }\left(\frac{1}{1-\varphi }\right)d\varphi $$

The Eq. (16) can be solved to get Eq. (17);

17 $${\left(\frac{{k}_{m}}{{k}_{eff,11}}\right)}^{2}={(1-\varphi )}^{({\beta }_{11}(1+\langle {\cos }^{2}\theta \rangle )+{\beta }_{33}(1-\langle {\cos }^{2}\theta \rangle ))}$$

The Eq. (17) can be solved iteratively for *k*_*eff,11*_. On the same lines, the Eq. (5) can be derived to give Eq. (18) that can also be solved iteratively for *k*_*eff,33*_.

18 $${\left(\frac{{k}_{m}}{{k}_{eff,33}}\right)}^{2}={(1-\varphi )}^{({\beta }_{11}(1-\langle {\cos }^{2}\theta \rangle )+{\beta }_{33}\langle {\cos }^{2}\theta \rangle )}$$

## Computational Material Design

The CPV systems should be able to serve the purpose of efficient production of electrical power for a reasonable lifetime. Based on the aforementioned requirements of the encapsulant, the TC should be increased, whereas CTE and G_∞_ should be decreased for the potential composite as compared with neat EVA and TPU. Setting the target values of TC, CTE and G_∞_ was established through optimization of material properties in a three-cell finite element (FE) model of the CPV module in the author’s previous work^27^. Wherein the FE model, governing equations, the life estimation-model, and material behaviors were completely elaborated. The material properties (TC, G_∞_, and CTE) for backside encapsulant were varied in the FE model and their effect on electrical power and module life was observed. The effect of increased TC appeared in the form of decreased cell temperature and hence increased electrical power. Similarly, the effect of decreased CTE and G_∞_ was to reduce thermal strains and stresses on the surrounding components including inter-cell connections, which were the critical regions. A sub-model was developed around the inter-cell connections regions to solve the problem for thermal stresses and strains in the critical regions with more accuracy. Finally, the module life was taken as the number of on-off cycles which could cause fatigue fracture of the inter-cell connections based on Morrow’s mean stress correction model for the strain life equation^31^. Conclusively from the parametric studies, it was determined that a TC of around 0.75 Wm^−1^K^−1^, CTE < 200 K^−1^ and G_∞_ < 0.2 MPa can provide both increased production of electrical power and module life of the CPV systems as compared with neat EVA. These are the target properties to be achieved by the computationally designed composite material in the present work.

### Selection of matrix

The encapsulant has to maintain its high electrical resistivity and has to achieve the target material properties. In addition, structural compatibility should not be affected. Therefore, different encapsulants used for the encapsulation of PV modules are selected as the potential matrices for the desired composite encapsulant. The material properties of these encapsulants are listed in Table 1. Among different encapsulants, PDMS and TPU are apparently the best candidates to reach the target range of ${G}_{\infty }$ based on their intrinsic material properties. However, to confirm this selection and elaborate the material design scheme, all candidate matrices are considered for the next steps of material design.

**Table 1:** Material properties of the prospective matrices for the composite encapsulant^40–44^.

Encapsulant Matrix	*k* (Wm^−1^K^−1^)	Poisson’s ratio	*G*_∞_ (MPa)	α (×10^−6^ K^−1^)
EVA	0.311	0.4	0.23	270
PVB	0.24	0.499	0.125	260
PDMS	0.15	0.44	0.0094	277
TPU	0.22	0.499	0.001124	190

### Selection of fillers

**Figure 1:**
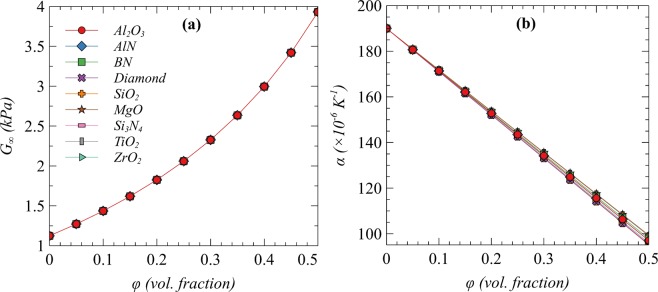
(**a**) *G*_∞_ and (**b**) *α* of TPU matrix composite as function of different filler’s volume fraction.

**Figure 2:**
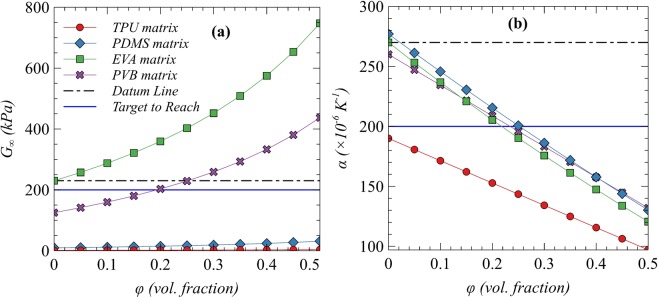
(**a**) *G*_∞_ and (**b**) *α* of composites with different matrices as function of volume fraction of alumina as filler.

To satisfy the requirements of the encapsulant, ceramic fillers are the potential candidates for the desired polymer composite. Due to the high electrical conductivity, metallic or carbon-based fillers are not suitable to be used as fillers and hence not considered in the current design. On the other hand, ceramic fillers can enhance both electrical resistivity and TC simultaneously owing to their very low electrical conductivity and high TC. Some potential ceramic fillers and their properties are listed in Table 2. The effect of fillers loading in the respective polymer matrix on ${G}_{\infty }$ and CTE is studied. Figure 1 shows the effect of filler’s volume fraction on ${G}_{\infty }$ and CTE of TPU as a matrix. It is found that increasing the volume fraction tends to increase ${G}_{\infty }$ and decrease CTE following the same trend regardless of the filler type. Hence, to achieve the target properties, any kind of listed ceramic filler can be used. However, the values of ${G}_{\infty }$ and CTE are mainly related to the type of matrix which affects the overall effective values as a function of filler’s loading as shown in Fig. 2, wherein the effect of adding Al_2_O_3_ on ${G}_{\infty }$ and CTE of all the matrices under consideration is presented.

**Table 2:** Bulk material properties of the prospective ceramic fillers^45,46^.

Filler material (pure)	*k* (Wm^−1^K^−1^)	Poisson’s ratio	*G* (MPa)	α (K^−1^)
Alumina (Al_2_O_3_)	33	0.21	88.0	4.40E-06
Aluminum Nitride (AlN)	177	0.23	126	4.30E-06
Boron Nitride (BN)	52	0.21	41.0	6.00E-06
Diamond	2000	0.2	440	8.00E-07
Fused Silica (SiO_2_)	1.5	0.15	31.0	5.50E-07
Magnesium Oxide (MgO)	60	0.35	92.0	9.00E-06
Silicon Nitride (Si_3_N_4_)	43	0.23	65.3	1.40E-06
Titania (TiO_2_)	11.8	0.27	90.0	8.40E-06
Zirconia (ZrO_2_)	2.7	0.22	53.4	2.30E-06

This behavior is attributed to the very large difference between the moduli and expansion coefficients of continuous (matrix) and discontinuous (fillers) phases. The effective properties such as CTE and G_∞_ of composites mainly depend upon the properties of the continuous phase (matrix). The comparative enhancement provided by different discontinuous phase particles (fillers) depends upon the relative numeric difference in the properties of matrix and fillers. The CTE of listed matrices is very high as compared with the ceramic fillers while the opposite is exact with G_∞_. Therefore, the comparative enhancement provided by different fillers is almost unchanged. Ceramic fillers have very large moduli (and very small CTEs) as compared with polymers. Therefore, upon application of mechanical (or thermal strain), the ceramic particles will never deform (or expand a little bit) and all the deformations would be localized to the matrix. The only strengthening mechanism is to restrict the motion of molecular chains at the interfacial boundaries and therefore, good interfacial strength is mandatory. Hence, it is observed that the type of matrix is the primary controlling factor for *G*_∞_ and CTE when the difference between the moduli of the matrix and fillers is very high^32,33^.

Based on the maximum limits of ${G}_{\infty }$ and CTE as highlighted in Fig. 2, EVA and PVB are not suitable candidate matrices as expected and hence can be omitted as far as ${G}_{\infty }$ and CTE are considered. However, further investigation is needed to see their effect on TC (to be discussed later). This investigation leads to the fact that TPU and PDMS are the most suitable matrices that could be used with any of the listed fillers for further material design. Although the target values of ${G}_{\infty }$ and CTE can be achieved using PVB as matrix at a filler volume content of 20 and 25%, respectively, selecting any of these two compositions cannot satisfy the requirements of both ${G}_{\infty }$ and CTE simultaneously. For the required target values of CTE in PVB matrix, a threshold content of 25% Al_2_O_3_ is required. However, significantly higher loading can lead to the value of ${G}_{\infty }$ that is expected to exceed the maximum limit, which is not recommended.

### Selection of filler attributes

Filler attributes are finalized based on the estimations of the effective TC model. It is discussed in detail by Raza *et al*.^34^ that two of the most sensitive parameters affecting the TC in polymer composites are the interface thermal resistance and the particle size of the fillers. These parameters play their role in the estimation of effective TC through a parameter *α* defined by Eq. (13), where the particle’s radius (*a*) is the decision-making parameter. Initially, the maximum allowed *R/a ratio* that can provide a reasonable enhancement of effective TC of the composite is determined, which depends upon the intrinsic TCs of phases (matrix and filler). The maximum *R/a ratio* indirectly identifies the minimum required particle radius (*a*). Based on the identified particle size, the final volume fraction of fillers is decided that can satisfy all the target values of the intended properties.

**Figure 3:**
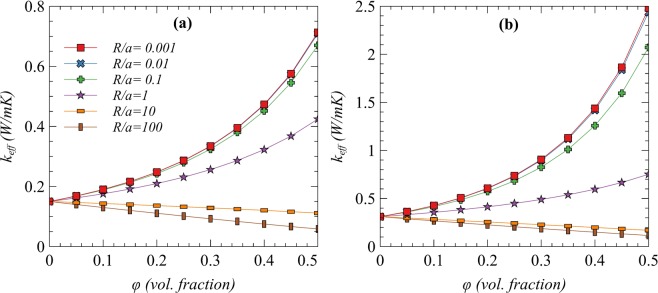
Estimation of effective thermal conductivity for the lowest and highest TCs combinations of matrices and fillers with different R/a ratios; (**a**) PDMS with spherical fused silica particulates (**b**) EVA with spherical diamond particulates.

To find the maximum allowed *R/a ratio*, two combinations of matrices and fillers with lowest and highest individual TCs are used to evaluate the effective TC of composites at different *R/a ratios* as shown in Fig. 3. The lowest TCs combination is fused silica with PDMS while the highest counterpart is diamond with EVA. It can be observed that the maximum allowed *R/a* ratio is close to unity in both of the combinations for a positive effect of fillers’ loading on effective TC. However, the enhancement of effective TC is reasonable with *R/a* = 0.1. The commonly reported value for interface thermal resistance (*R*) between polymer matrices and ceramic fillers is around 3 × 10^−7^ m^2^KW^−1^ ^35,36^ which results in a minimum required particle’s radius of 3 μm i.e. a particle size of 6 μm.

**Figure 4:**
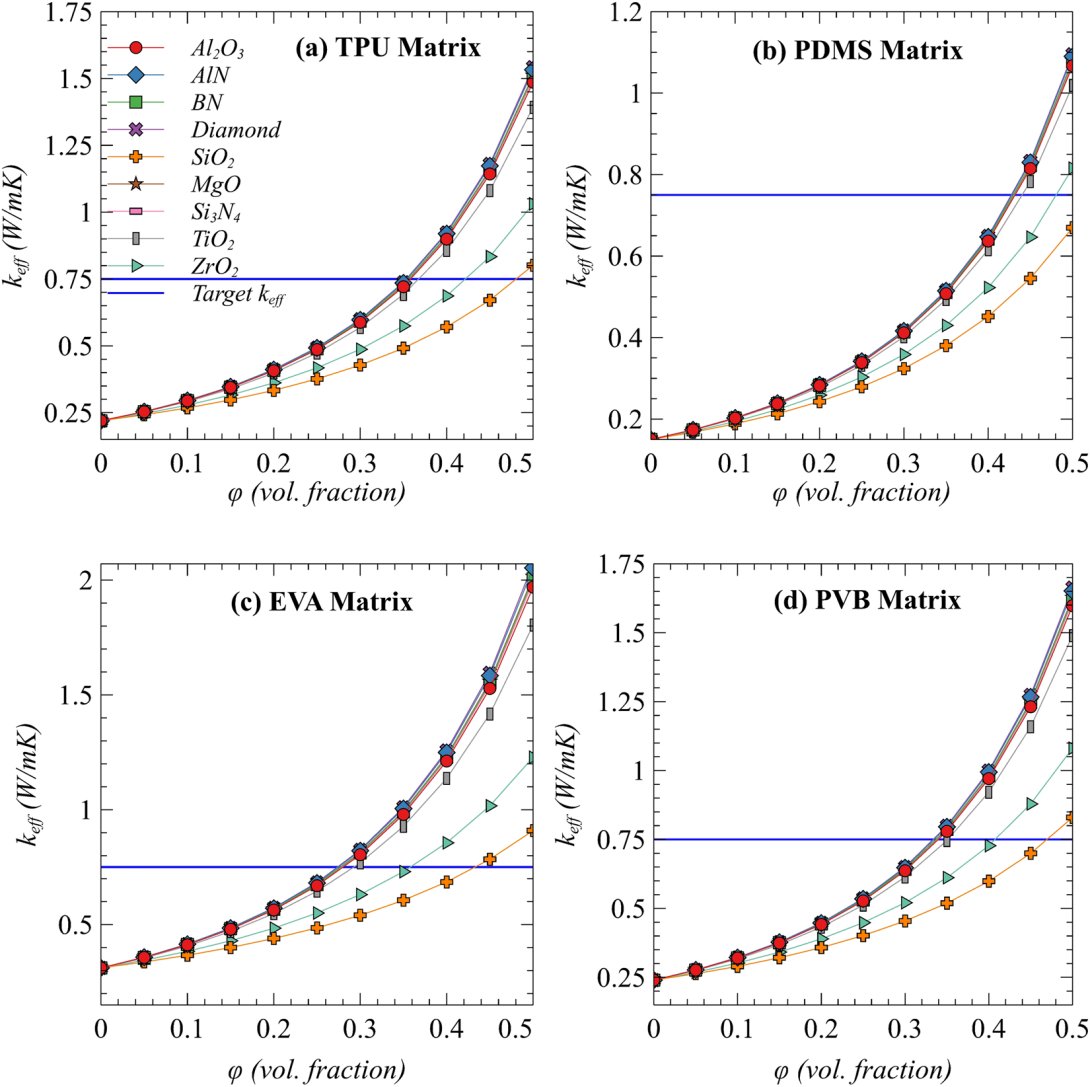
Estimation of effective thermal conductivities in different matrices and with different fillers at fixed R/a = 0.1.

After identification of minimum particle size, all candidate matrices and fillers are used to predict the effective TC. The winner candidates and their required volume fractions that can satisfy all the targets of thermal and mechanical properties are finalized based on the estimations of effective TC with the identified threshold particle size of fillers. Figure 4 presents effective TC as a function of volume fraction in all possible combinations of listed matrices and fillers with *a* = *3 μm*. It is depicted from Fig. 4 that a PDMS as a matrix and ZrO_2_ or SiO_2_ as a filler are not recommended keeping in view the fact that a very high filler loading would be required to reach the target effective TC of 0.75 Wm^−1^K^−1^. On the contrary, the other fillers can provide nearly equal improvement in TC due to the converging effect of *k*_*f*_^34,35^. The same converging effect due to the large difference between the properties of matrices and fillers has been explained in the discussion of CTE and G_∞_ above. For further shortlisting of the fillers, one has to on make other criteria such as cost per unit volume of the fillers, availability, possible reactivity with the matrix or the other components of PV modules, etc. The desired target of TC is expected to be achieved with a volume fraction of 0.3 in EVA and 0.35 in TPU and PVB matrices with spherical particles. Moreover, larger particle size, particles with high aspect ratio or hybrid fillers can be used to decrease the required volume fraction^37^.

Decisively, from the material design scheme, EVA and PVB are not recommended as far as the required values of ${G}_{\infty }$ and CTE are concerned. Similarly, PDMS as a matrix and ZrO_2_ and SiO_2_ as fillers are eliminated by the TC model owing to their lower intrinsic TC. All of the other candidate fillers (as listed in Table 2) are capable to provide the desired target values of TC, ${G}_{\infty }$ and CTE with a volume fraction of around 0.35 and a particle size of 6 μm. However, for the development of composites Al_2_O_3_ and AlN are considered. Al_2_O_3_ has low TC and low shear modulus while AlN is the second-highest after diamond among the listed fillers in Table 2. The correspondence between the experimental measurements and computational design with these low and high –profile fillers will establish the validation and confidence in computational material design. Based on the available particle size and approximate shape morphology, the required volume fraction is re-estimated for the TPU matrix with Al_2_O_3_ and AlN as summarized in Table 3. The required estimated volume percent for the available Al_2_O_3_ and AlN is 22.5 and 23%, respectively.

**Table 3:** Results of Material design scheme for TPU matrix with the available particle sizes.

Matrix	Filler	Average size parameters		Estimated volume fraction	Experimentally mixed volume fraction	Name
		Radius = a_1_	p			
TPU	AlN	3 μm	1.67	0.230	0.20	S1
TPU	Al_2_O_3_	3 μm	2	0.225	0.20	S2
TPU	AlN	3 μm	1.67	0.230	0.25	S3
TPU	Al_2_O_3_	3 μm	2	0.225	0.25	S4

## Materials and Methods

**Figure 5:**
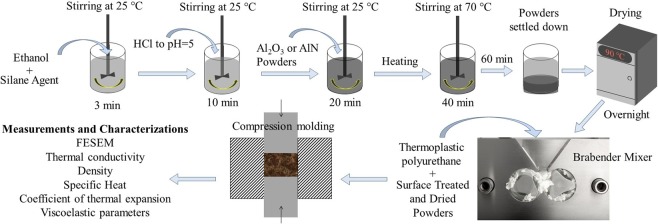
Schematic diagram for the route of composite sample’s development.

The development of composites includes all the steps starting from raw materials to the characterization and measurement of material properties. A schematic flow chart of the steps involved in the development of composites is shown in Fig. 5.

### Materials

Al_2_O_3_ and Surface treating agent γ-methylacryloxypropyl trimethoxy silane were purchased from Sigma-Aldrich and AlN powders from Surmet Corporation (USA). TPU was supplied by Taiwan PU Corporation. The information about the particle size has been listed in Table 3.

### Surface treatment

The ceramic particles were surface treated before mixing with TPU to enhance interfacial compatibility. γ-methylacryloxypropyl trimethoxy silane equivalent to 2% by weight of the filler was mixed with ethanol for 3 minutes at room temperature. Then pH was adjusted to 5 by dropwise adding dilute HCl and stirred for another 10 minutes at room temperature. For surface treatment, initially, pre-weighed amounts of Al_2_O_3_ or AlN powder was added to the solution and stirred for 20 min at room temperature. The mixture was then heated to 70 °C on a hot plate with continued stirring for another 40 minutes. Heating and stirring were stopped and the particles were allowed to settle down for 1 hour. Finally, the solution was poured out and the settled particles were dried overnight at 90 °C in a vacuum oven. The surface-treated powders were stored in vacuum desiccators to avoid any moisture or dust contamination.

### Melt-mixing

Before mixing, the TPU granules were dried by placing them in a vacuum desiccator overnight. The pre-treated ceramic powders and TPU granules were mixed in molten state using Brabender Mixer. After the three zones of mixing chamber had attained the processing temperature of 150 °C, the dried TPU granules and the ceramic powders were added into the mixing chamber at a slower rpm (30 rpm) and the chamber was closed. The mixing speed was then increased to the processing speed of 60 rpm and continued for 20 minutes. After each mixing cycle, the chamber was cleaned thoroughly to avoid contamination to the next compositions. The output of the Brabender Mixer is in the form of irregular lumps that cannot be used for any type of testing or characterization. So these composite lumps are molded to make samples of the desired shapes for testing.

### Compression molding

A pre-weighed amount of the lumps, depending upon the required thickness was hot-pressed in a compression-molding machine at 120 °C. The produced samples were cylindrical (ϕ 31 mm and as desired thickness) that could be cut further to get the desired shape for a particular test.

### Testing/Characterization

Measurement of TC, *G*_∞_, and CTE is required to validate the material design approach. However, the other properties like heat capacity, density, and viscoelastic parameters were also evaluated to be used as inputs in sequentially coupled FE model of a three-cell structure. Moreover, the microstructures of developed composites examined using FESEM. The machines used for different characterizations are listed in Table 4.

**Table 4:** List of equipment used for used in testing and characterization of synthesized samples.

Property to be evaluated	Used equipment/machine
Long term shear modulus and viscoelastic behavior	Mettler Toledo DMA/SDTA 1+ - Dynamic Mechanical Analyzer
Coefficient of thermal expansion	Mettler Toledo TMA/SDTA 2+ - Thermo Mechanical Analyzer
Thermal Conductivity	TCi Thermal Conductivity Analyzer by C-Therm Technologies Ltd., Canada
Heat capacity at constant pressure	Mettler Toledo DSC 3+ - Differential Scanning Calorimeter
Density	Archimedes Principle
Microstructural examination	FESEM Tescan Lyra 3

## Results and Validation

### Microstructural examination

**Figure 6:**
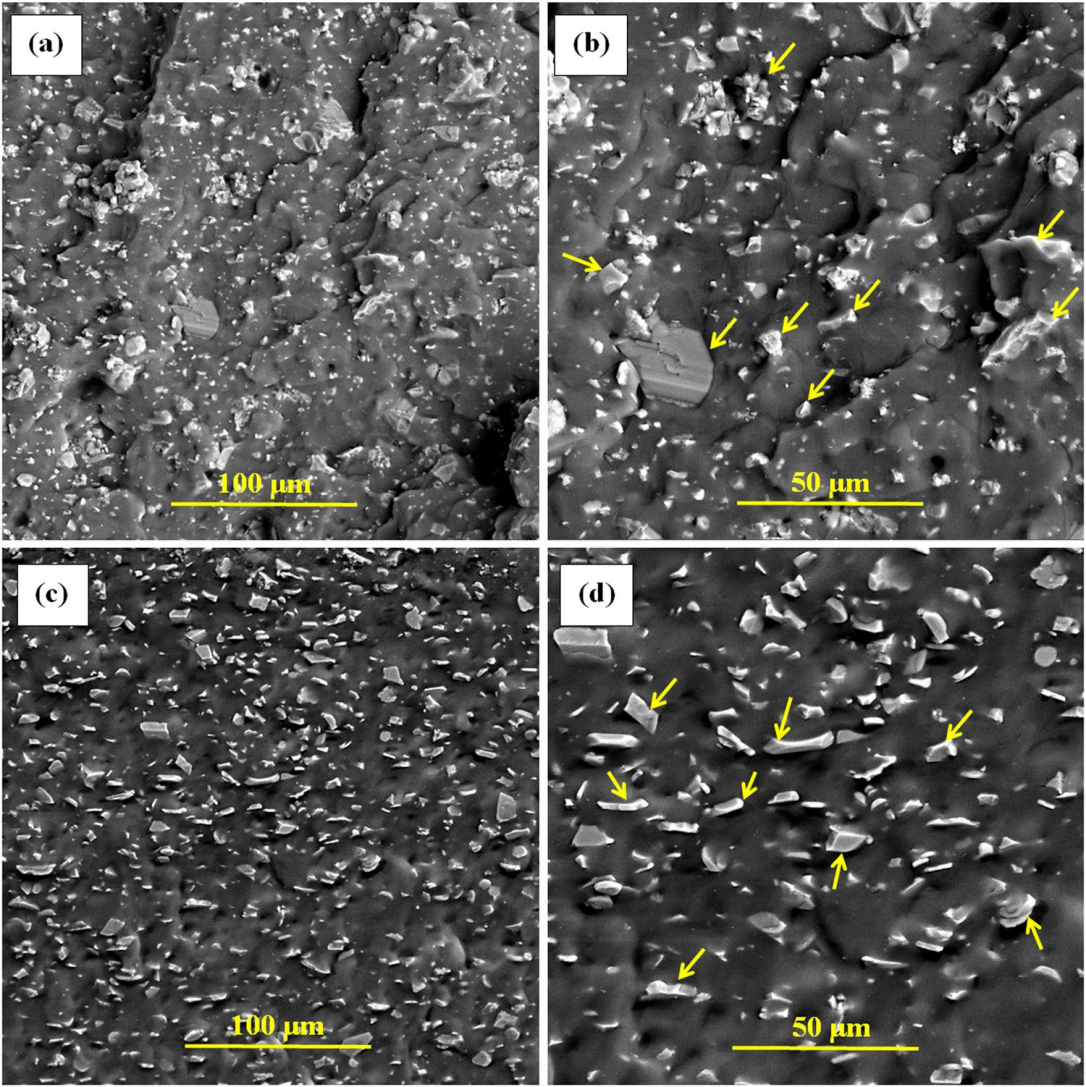
FESEM fractured surface of samples S1 (**a**) & (**b**) and S4 (**c)** & (**d**) taken at different magnifications; the arrow marks indicate the AlN particles in (**b**) and Al_2_O_3_ particles in (**d**).

The distribution of ceramic particles in the microstructure was observed to evaluate the effectiveness of the melt-mixing and compression molding synthesis route. The compression-molded samples were brittle-fractured after placing them in a chiller at −115 °C and the fractured surfaces were gold-coated for electron imaging. The imaging was conducted in backscattered mode to have a better contrast of ceramic vs. polymers. The fractured surfaces of samples S1 and S4 are shown in Fig. 6. No porosity is observed and the particles are distributed very well which indicates that the synthesis route was good enough to mix the ingredients.

### Material properties

The estimated and measured thermo-physical properties of the produced samples are listed in Table 5. A reasonable agreement between the measured and estimated TCs and CTEs is observed. Similarly, the measured densities and specific heats of the samples are also close to the theoretical values indicating almost fully densified samples supported by the FESEM observations. The higher values of TCs and lower values of CTEs in the samples S3 and S4 are due to higher volume fractions of filler comparatively. AlN has a higher intrinsic TC than Al_2_O_3_, therefore the samples S1 and S3 are expected to possess higher TCs, but the unexpected results of TC values are dedicated to the disc-like shape of Al_2_O_3_ particles used in this work.

**Table 5:** Measured thermal and physical properties of the samples listed in Table 3.

Name	Effective TC = k_eff_ (Wm^−1^K^−1^)		Effective CTE = α_eff_ (*10^−6^K^−1^)		*ρ* (gcm^−3^)	C_p_ (Jkg^−1^K^−1^)
	Estimated	measured	estimated	Measured	Measured	measured
S1	0.72	0.703	160	180	1.605	1290
S2	0.72	0.718	160	192	1.721	1055
S3	0.78	0.768	136	150	1.705	1240
S4	0.78	0.793	136	158	1.881	1025

**Figure 7:**
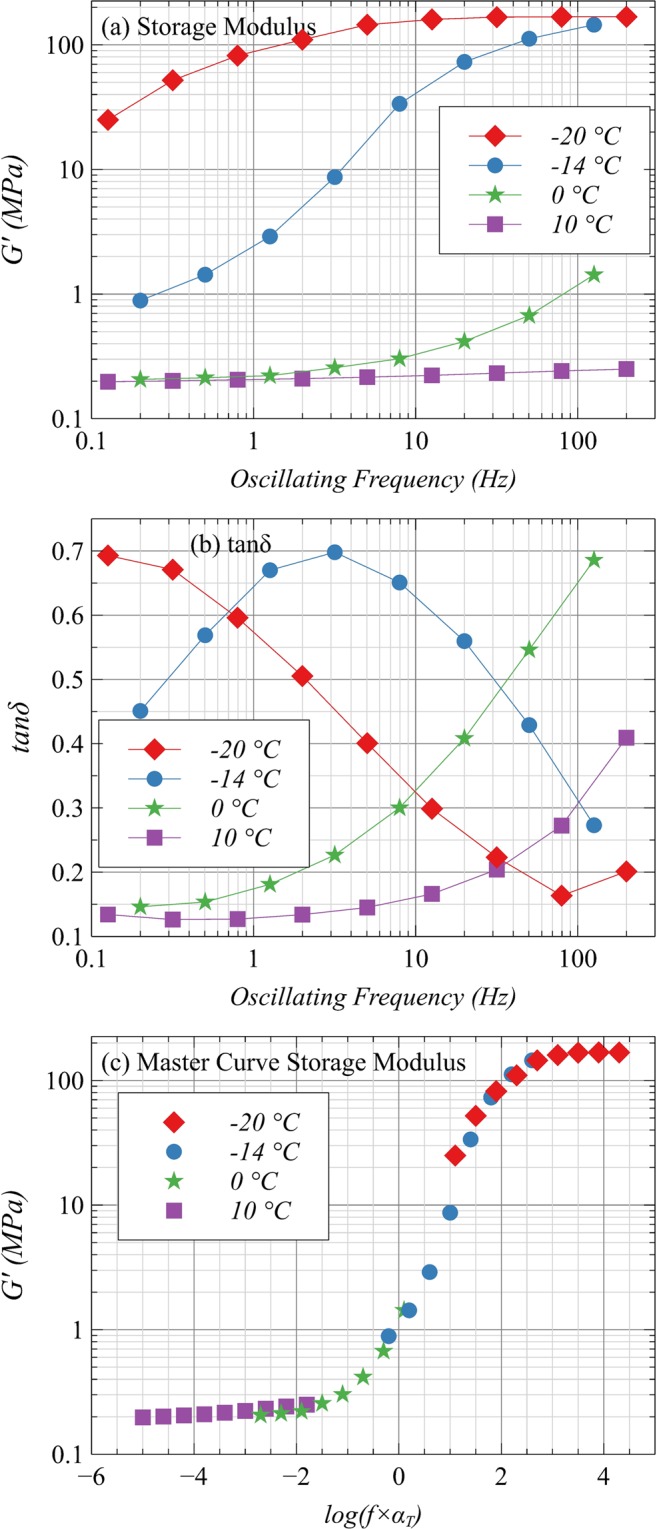
(**a**) The storage modulus (G′) and (**b**) tanδ of S1 measured as function of temperature and oscillating frequency; (**c**) Master Curve of Storage Modulus for S1 generated for T_ref_ = −11 °C by horizontal shifting of the measured curves.

The sample S1 was selected for the measurement of *G*_∞_ and viscoelastic parameters using dynamic mechanical analyzer (DMA). Cuboid samples with a geometry factor of at least 30 as recommended by the manufacturer were used for DMA measurements. The samples were cooled to −20 °C using liquid nitrogen. At different temperatures, isotherms of storage modulus (*G*′) and tanδ were generated as a function of oscillating frequency ranging from 0.1 to 200 Hz (Fig. 7(a,b) for *G*′ and tanδ respectively). For each isotherm, the system was allowed to settle for 5 minutes to homogenize temperature in the entire sample. Typical behavior of viscoelastic polymers is observed in the sample i.e. a stiffer response at a higher oscillating frequency and vice versa.

**Figure 8:**
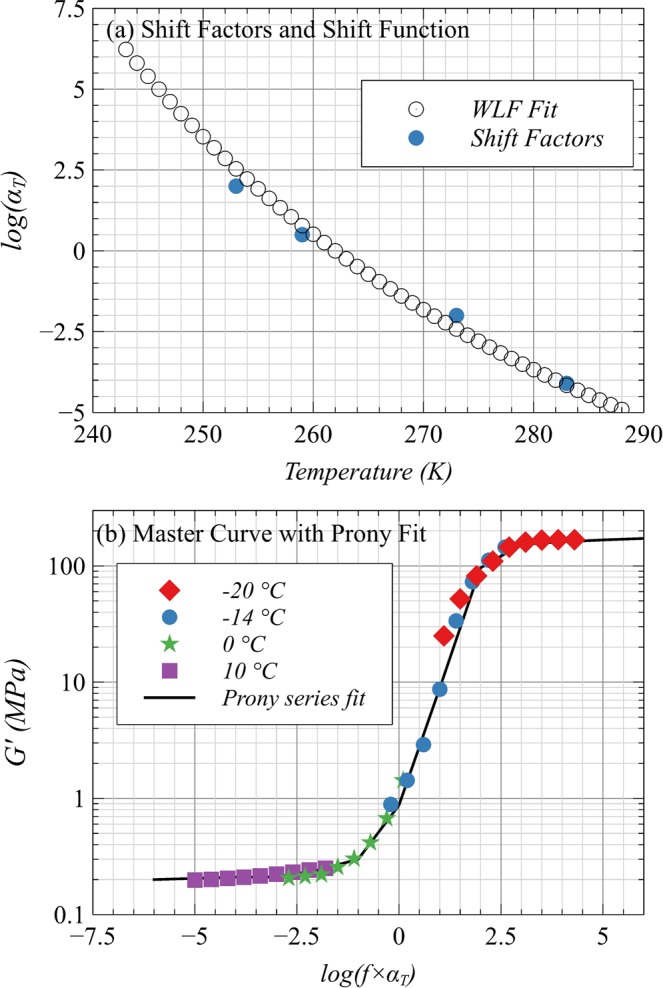
(**a**) Shift factors plot and WLF fit of Storage Modulus Master Curve. (**b**) Prony series fit of Storage Modulus data for S1 at T_ref_ = −11 °C.

The curves are similar to “thermo-rheologically simple” polymers and therefore, the master curve of *G*′ as a function of a wide range of frequency can be generated by horizontal shifting of the curves. The curves of *G*′ were horizontally shifted on a log scale to make a continuous curve for *T*_*ref*_ = −11 °C which is the glass transition temperature of TPU (for the particular grade used in this study). The applied shift factors (a_T_) for −20, −14, 0 and 10 °C were 2, 0.5, −2 and −4.1, respectively. The corresponding master curve of *G*′ is shown in Fig. 7(c) that makes a continuous curve, which can be simplified by Generalized Maxwell Model for linear viscoelastic materials^38,39^. The Williams–Landel–Ferry equation (WLF) constants of shift factor determined by linear curve fitting are C_1_ = 20 and C_2_ = 80 K. In Fig. 8(a), the values of actually used shift factors are compared to the WLF shift function which is close-fitting. Finally, Prony series coefficients for 9-term Generalized Maxwell Model are determined using an error minimization code in MATLAB (Table 6) and the resulting Prony fit is compared to the master curve of *G*′ in Fig. 8(b). The parameters listed in Table 6 and the constants of WLF shift function were used as inputs to model the thermomechanical (constitutive) behavior of developed composite and finally to evaluate the performance in the finite element model already published^27^.

**Table 6:** The 9-Term Coefficients of Prony Series for S1 at T_ref_ = −11 °C.

Branch #	G_i_ (MPa)	τ_i_ (sec)
Pure elastic	*G*_∞_ = 0.2	—
1	30	1.00E − 09
2	10	1.00E − 06
3	12	1.00E − 04
4	60	5.00E − 03
5	90	3.00E − 02
6	4	1.00E − 02
7	1	1.00E + 00
8	0.08	8.00E + 01
9	0.01	1.00E + 05

## Performance Evaluation

The measured properties of the sample S1 (TPU + 20% AlN) were used in a finite element model of a representative three-cell module to estimate the electrical power and module life and compared with neat encapsulants, TPU and EVA. The details of the finite element model such as material behaviors, governing equations, boundary conditions, and solution strategy have been published already^27^. The TC and specific heat were the inputs to the heat transfer study while the other properties were inputs to the thermal stress and strain study and finally, the obtained results are summarized in Table 7. The highest cell temperature with neat TPU is attributed to its lowest TC as compared with neat EVA and proposed composite. Due to its highest TC, the proposed composite is capable to offer the lowest cell temperature and hence enhanced electrical power by 4.3% when compared with neat TPU. The highest mean stress and strain range are imposed by EVA because its CTE and G_∞_ values are the highest while the opposite is exact with neat TPU. The estimated life of 17.8 years with the proposed composite is good enough to pay back with a reasonable profit. On the other hand, the estimated life of ~61 years with TPU is too high to cope with the advancement of technology and degradation caused by other factors.

**Table 7:** Results of performance evaluation using the properties sample S1 in the finite element model.

Name of the parameter	Magnitudes obtained			
	Using neat TPU	Using neat EVA	Using proposed composite (S1)	
Cell Temperature	68.8 °C	66.1 °C	63 °C	
Electrical Power	185 W	189 W	193 W	
% improvement in power output as compared with neat polymers	—	—	TPU	EVA
			4.3%	2.1%
Mean stress	37.5 MPa	44.4 MPa	44.3 MPa	
Strain range	0.004	0.00593	0.00542	
Estimated fatigue life	61.3 Years	12.7 Years	17.8 Years	

## Conclusions

A computational approach is presented for the design of high-performance polymer composites for the backside encapsulant of CPV systems. The material design is carried out using property estimation models followed by validation and performance evaluation. The design starts with selecting performance indicators and critical material properties. For CPV systems, electrical power and module life are considered as performance indicators, and thermal conductivity, coefficient of thermal expansion, and long-term shear modulus are considered as critical material properties. Based on target properties as predicted from FE-based performance evaluation, various candidate polymer matrices and fillers are considered at the material design stage. Thermoplastic polyurethane as matrix and ceramics (such as Al_2_O_3_ and AlN) as fillers are the best among various candidates. For validation of the design, some representative composites are synthesized and characterized. The properties such as thermal conductivity, coefficient of thermal expansion, viscoelastic behavior and long-term shear modulus are measured which are found in close agreement with the predictions. Moreover, the parameters of 9-term Generalized Maxwell Model and WLF shift function are determined by generating the master curve method. The performance of the module with the designed composite as encapsulant is assessed by using the measured properties in the finite element model for a three-cell CPV structure. The proposed TPU-composite is more capable and results in a temperature drop of 5.8 and 3.1 °C leading to an increased electrical power by 4.3 and 2.1% when compared with neat TPU and EVA, respectively along with a more practical module life. The presented computational design and development strategy can serve as a useful guideline for composite manufacturers in developing new potential polymer composites for target applications.

## Supplementary information

Supplementary Information.
