# Supplementary material for: Computational design and development of high-performance polymer-composites as new encapsulant material for concentrated PV modules
Source: Sci Rep. 2020 Mar 24;10:5304. doi: 10.1038/s41598-020-62191-9 (PMC7093474; doi:10.1038/s41598-020-62191-9)
Supplement: Supplementary file 1 — Supplementary Information. [file 41598_2020_62191_MOESM1_ESM.docx]

**Computational design and development of high-performance polymer-composites as new encapsulant material for concentrated PV modules**

Kabeer Raza^a^, Syed Sohail Akhtar^a,b,*^Abul Fazal M. Arif ^c^ , Abbas Saeed Hakeem^b^

^a^Mechanical Engineering Department, King Fahd University of Petroleum & Minerals (KFUPM), Dhahran, Saudi Arabia

^b^Center of Excellence in Nanotechnology, King Fahd University of Petroleum & Minerals, Dhahran, Saudi Arabia

^c^Department of Mechanical Engineering, McMaster Manufacturing Research Institute, McMaster University, Hamilton, Canada

Email of the Corresponding author: [ssakhtar@kfupm.edu.sa](mailto:ssakhtar@kfupm.edu.sa)

# Supplementary Information

**Derivation of Equation (14) for small volume fractions**

Equation (4) can be simplified for infinitesimal values of φ by rationalization i.e. multiplying and dividing by the conjugate of the denominator;

Where the terms with φ^2^ become zero to very small multiplying factor. Further simplification yields;

Where some of the terms are canceled due to opposite signs (such as $\beta_{11}^{i}L_{11}^{i}$) and yields the following equation, which is further simplified leading to equation (14);
